# Age-Dependent FOSB/ΔFOSB Response to Acute and Chronic Stress in the Extended Amygdala, Hypothalamic Paraventricular, Habenular, Centrally-Projecting Edinger-Westphal, and Dorsal Raphe Nuclei in Male Rats

**DOI:** 10.3389/fnagi.2022.862098

**Published:** 2022-05-03

**Authors:** László Ákos Kovács, Nóra Füredi, Balázs Ujvári, Abolfazl Golgol, Balázs Gaszner

**Affiliations:** ^1^Department of Anatomy, Research Group for Mood Disorders, Medical School, University of Pécs, Pécs, Hungary; ^2^Center for Neuroscience & Szentagothai Research Center, Pécs University, Pécs, Hungary

**Keywords:** immunohistochemistry, deltaFosB, restraint, chronic mild stress, BNST

## Abstract

FOS proteins are early-responding gene products that contribute to the formation of activator protein-1. Several acute and chronic stimuli lead to Fos gene expression, accompanied by an increase of nuclear FOS, which appears to decline with aging. FOSB is another marker to detect acute cellular response, while ΔFOSB mirrors long-lasting changes in neuronal activity upon chronic stress. The notion that the occurrence of stress-related mood disorders shows some age dependence suggests that the brain’s stress sensitivity is also a function of age. To study age-dependent stress vulnerability at the immediate-early gene level, we aimed to describe how the course of aging affects the neural responses of FOSB/ΔFOSB in the acute restraint stress (ARS), and chronic variable mild stress (CVMS) in male rats. Fourteen brain areas [central, medial, basolateral (BLA) amygdala; dorsolateral- (BNSTdl), oval- (BNSTov), dorsomedial-, ventral- (BNSTv), and fusiform- (BNSTfu) divisions of the bed nucleus of the stria terminalis; medial and lateral habenula, hypothalamic paraventricular nucleus (PVN), centrally-projecting Edinger-Westphal nucleus, dorsal raphe nucleus, barrel field of somatosensory cortex (S1)] were examined in the course of aging. Eight age groups [1-month-old (M), 1.5 M, 2 M, 3 M, 6 M, 12 M, 18 M, and 24 M] of rats were exposed to a single ARS vs. controls. In addition, rats in six age groups (2, 3, 6, 12, 18, and 24 M) were subjected to CVMS. The FOSB/ΔFOSB immunoreactivity (IR) was a function of age in both controls, ARS- and CVMS-exposed rats. ARS increased the FOSB/ΔFOSB in all nuclei (except in BLA), but only BNSTfu, BNSTv, and PVN reacted throughout the examined lifespan. The CVMS did not increase the FOSB/ΔFOSB in BLA, BNSTov, BNSTdl, and S1. PVN showed a constantly maintained FOSB/ΔFOSB IR during the examined life period. The maximum stress-evoked FOSB/ΔFOSB signal was detected at 2–3 M periods in the ARS- and at 6 M, 18 M in CVMS- model. Corresponding to our previous observations on FOS, the FOSB/ΔFOSB response to stress decreased with age in most of the examined nuclei. Only the PVN exerted a sustained age-independent FOSB/ΔFOSB, which may reflect the long-lasting adaptation response and plasticity of neurons that maintain the hypothalamus-pituitary-adrenal axis response throughout the lifespan.

## Introduction

The FOS proteins are products of immediate early genes (IEGs), and their expression can be induced rapidly and transiently by various external stimuli (Sheng and Greenberg, 1990; Gass et al., 1992). FOS family members, including FOS, FOSB, ΔFOSB, FRA1, and FRA2, create heterodimers with JUN proteins, such as JUNC, JUNB, and JUND, to form the transcription factor activator protein-1 (AP-1) binding to AP-1 sites (Saffen et al., 1988; Zerial et al., 1989; Hope et al., 1992; Okuno, 2011). The AP-1 site is an abundantly represented sequence (TGACTCA) in the promoter regions of genes, thereby the AP-1 is involved in the regulation of several cellular processes, such as proliferation, differentiation, and apoptotic cell death (Herdegen and Leah, 1998; Okuno, 2011; Gazon et al., 2018). A large number of genes are affected by the FOS proteins and the AP-1 are well-known in the fields of cancer research, inflammation, immunity, and bone development (Bozec et al., 2010; Gazon et al., 2018; Bejjani et al., 2019). However, our knowledge is limited to the functional significance of FOS, FOSB, and ΔFOSB in the central nervous system (Okuno, 2011; Nestler, 2015; Eagle et al., 2018; You et al., 2018).

The expression of Fos genes has been extensively studied both at mRNA and protein levels in the central nervous system (Curran and Morgan, 1987; Bullitt, 1990; Hope et al., 1992; Kovács, 1998, 2008; Alibhai et al., 2007). *Fos*, *FosB*, *ΔFosb*, and *Fra2* mRNAs can be detected minutes after the supra-threshold stimulus (Honkaniemi et al., 1994; Kovács, 1998; Alibhai et al., 2007). The fastest dynamics is characteristic for *Fos* with maximal mRNA level 30 min after the stimulus. *FosB* peaks at 60 min, while *ΔFosB* reaches the highest mRNA level 180 min after the onset of the stimulus (Kovács, 1998, 2008; Alibhai et al., 2007; Nestler, 2015). FOS products peak at protein level within a few hours: FOS (1.5–2 h); FOSB (2–3 h); ΔFOSB, FRA1, and FRA2 (2–6 h) (Acquaviva et al., 2001; Carle et al., 2007; Nestler, 2015). These proteins disappear within 8–12 h after the onset of the stimulus, unlike ΔFOSB, a splice variant of FOSB that remains detectable up to a day (Kovács, 1998; Acquaviva et al., 2001; Carle et al., 2007; Nestler, 2015). In the case of repeated stimuli, neurons exhibit partial or complete adaptation, which reflects diminished or absent FOS and FOSB signals (Melia et al., 1994; Stamp and Herbert, 1999; Viau and Sawchenko, 2002). Nevertheless, the neuronal ΔFOSB response usually does not attenuate upon repeated stimuli (e.g., cocaine administration, social defeat, and immobilization) or chronic stress, but this phenomenon is highly brain area-specific (Hope et al., 1992; McClung et al., 2004; Vialou et al., 2015; Kovács et al., 2019). In contrast to FOS and FOSB, the ΔFOSB can be accumulated during repeated exposure to the stimulus (e.g., chronic stress) and at the protein level, it remains detectable even a week after the last exposure (Nestler, 2015). The basal expression of FOS, FOSB, and ΔFOSB in the central nervous system is relatively low with some region specificity (Herdegen et al., 1995; Kellogg et al., 1998; Perrotti et al., 2004; Kovács et al., 2018, 2019). Due to their rapidly inducible expression, the detection of these proteins is a powerful and frequently used technique in functional-morphological studies on stress-recruited brain areas (Kovács, 2008; Okuno, 2011).

The paraventricular nucleus of the hypothalamus (PVN) harbors corticotropin-releasing hormone (CRH)-producing cells, which play a critical role in the stress responses orchestrated by the hypothalamus-pituitary-adrenal (HPA) axis. CRH released from PVN stimulates adrenocorticotropic hormone (ACTH) secretion from the anterior pituitary which controls the glucocorticoid secretion in the cortex of the suprarenal gland (Herman et al., 2016; Deussing and Chen, 2018).

Acute stress, such as immobilization (Imaki et al., 1995), pain (Rouwette et al., 2011), ether inhalation (Kovács and Sawchenko, 1996), or a single dose of cocaine (Chocyk et al., 2006) increases the level of CRH or *Crh* mRNA, which is accompanied by the presence of FOS and/or FOSB/ΔFOSB products (Kovács and Sawchenko, 1996; Viau and Sawchenko, 2002; Rouwette et al., 2011; Kovács et al., 2019). CRH neurons co-express FOS products (Kovács and Sawchenko, 1996; Yanagita et al., 2007; Kovács et al., 2019) and the AP-1 sites are present in the Crh gene promoter (Malkoski and Dorin, 1999). Therefore, it seems feasible that AP-1 is involved in the regulation of *Crh* expression. Nevertheless, after a stimulus, *Crh* hnRNA increases earlier than *Fos/FosB* mRNAs and FOS proteins suggesting that AP-1 does not have a direct short-term regulatory role in Crh gene expression (Kovács and Sawchenko, 1996; Alibhai et al., 2007). In contrast to *in vivo*, *in vitro* experiments on hypothalamic cells denote that FOSB may be involved in the long-lasting upregulation of Crh gene expression (Kageyama et al., 2014). Chronic variable mild stress (CVMS) (Willner, 2005, 2016; Sterrenburg et al., 2011; Kovács et al., 2019), chronic opioid administration (García-Pérez et al., 2012), or chronic social defeat stress (Vialou et al., 2015) result in an increased level of FOSB/ΔFOSB in the PVN. Upon CVMS, increased FOSB/ΔFOSB immunoreactivity was associated with heightened CRH immunosignal in the PVN (Kovács et al., 2019). Disturbances of the HPA axis (Nemeroff, 1988; deKloet et al., 2016) and other extra-hypothalamic CRH-producing centers (Regev et al., 2012) are frequently associated with stress-evoked psychiatric disorders, including major depressive disorder (Nemeroff, 1988; Bangasser and Kawasumi, 2015), anxiety (Stenzel-Poore et al., 1994), posttraumatic stress disorder (Bremner et al., 1997), and schizophrenia (Lammers et al., 1995). Therefore, it is essential to gain deeper insight into the control of PVN/CRH and its higher-order regulation (Sparta et al., 2013; Herman et al., 2016).

The amygdaloid complex is comprised of heterogeneous subdivisions. They receive, convert, and process sensory inputs, such as olfactory stimuli in the medial amygdala (MeA) (Keshavarzi et al., 2015). The basolateral division (BLA) is involved in cognitive processes (e.g., fear and memory), while the central nucleus (CeA) participates in central stress response by its CRH-producing cells (Merchenthaler, 1984; Partridge et al., 2016; Sun et al., 2020). Interestingly, the MeA, BLA, and CeA do not show an increase in FOSB/ΔFOSB in response to acute restraint stress (Kovács et al., 2019; Kang et al., 2021), or chronic variable/repeated stress (Stamp and Herbert, 2001; Sterrenburg et al., 2011; Kovács et al., 2019), but they all display a strong FOS response to acute restraint stress (ARS) (Kovács et al., 2018). The nuclei of the amygdaloid complex that contribute to the control of PVN *via* centers are now considered part of the extended amygdala (Dong et al., 2001; Ulrich-Lai and Herman, 2009; Sparta et al., 2013).

The bed nucleus of the stria terminalis (BNST) is composed of several sub-nuclei (Dong et al., 2001; Hammack et al., 2010). The posterior BNST inhibits, while anterior BNST stimulates, the PVN/CRH (Choi et al., 2007; Ulrich-Lai and Herman, 2009). The oval division of BNST (BNSTov) is characterized by high basal FOSB/ΔFOSB content (Sterrenburg et al., 2011; Kovács et al., 2019), but does not display increased FOSB/ΔFOSB in stressful situations such as ARS or CVMS. In contrast to the BNSTov, the fusiform nucleus of BNST (BNSTfu) responds with increased FOSB/ΔFOSB after CVMS exposure (Sterrenburg et al., 2011). Besides the PVN, the CeA, BNSTov, and BNSTfu contain large populations of CRH-immunoreactive cells (Merchenthaler, 1984; Nguyen et al., 2016).

The habenular complex is an important limbic center involved in mood control (McLaughlin et al., 2017; Metzger et al., 2017) that is divided into a medial- (MHb) and lateral nucleus (LHb). The downregulation of genes (e.g., choline acetyltransferase) involved in MHb cholinergic signaling was found to be associated with anhedonia-like behavior and suicide (Han et al., 2017). Considerable basal FOSB/ΔFOSB immunoreactivity was detected both in LHb and MHb that increased upon chronic unpredictable stress in the LHb (Zhang et al., 2018).

The centrally-projecting Edinger-Westphal nucleus (EWcp) harbors urocortin1-producing neurons (Kozicz et al., 1998; Ryabinin et al., 2005). They show FOS and FOSB/ΔFOSB neuronal activity in various acute and chronic stress models in rodents (Gaszner et al., 2004; Turek and Ryabinin, 2005; Rouwette et al., 2011; Kormos et al., 2016, 2022; Farkas et al., 2017; Kovács et al., 2018). The alteration of urocortinergic neuronal function affects mood status (Ujvári et al., 2022, for review, see Kozicz et al., 2011) and it is associated with suicide (Kozicz et al., 2008).

Dorsal raphe nucleus (DR) serotonergic (5-HT)-neurons play well-characterized roles in stress adaptation and stress-related depressive disorders (Hernández-Vázquez et al., 2019; Ohmura et al., 2020). The DR neurons show FOS and FOSB/ΔFOSB activation both in acute and chronic stress in rodents (Ishida et al., 2002; Turek and Ryabinin, 2005; Kormos et al., 2016; Farkas et al., 2017; Kovács et al., 2018; Lopes et al., 2019; Nascimento et al., 2020).

Only a few studies were published on the stress-evoked IEGs at various age periods, but they focused mainly on the FOS response (Kitraki et al., 1993; Kellogg et al., 1998; Viau et al., 2005; Romeo et al., 2006; Meyza et al., 2007). Their limitations are as follows: (a) only a few (1–3) periods of life, especially the late adolescence [2 months-of age (M)], early adulthood (3 M), and old age (24 or 30 M) were compared. Moreover, (b) only a few of them mapped multiple brain areas (Kellogg et al., 1998; Meyza et al., 2007; Kovács et al., 2018).

These studies concluded that the basal *Fos* mRNA expression and FOS protein content did not change in senescence (Desjardíns et al., 1997). In seizure-induced stress, aged (20, 21, and 28 M) animals displayed delayed *Fos* mRNA response and decreased FOS content, compared to the younger (3, 5, and 6 M) groups (D’Costa et al., 1991; Retchkiman et al., 1996; Nagahara and Handa, 1997). Studies on ARS-evoked FOS response and plasticity found that 1 M animals showed increased FOS content compared to the young adults (2–2.5 M) in the PVN (Viau et al., 2005; Romeo et al., 2006). However, Kellogg et al. (1998) reported that more brain regions were activated by stress exposure in young adults than in 1 M animals.

In our earlier studies, we found a decreasing FOSB/ΔFOSB immunoreactivity in CeA- and BNSTov/CRH neurons during aging (Kovács et al., 2019). Contrary to amygdala nuclei, the PVN/CRH neurons showed a robust FOSB/ΔFOSB expression in both ARS- and CVMS-exposed young rats that sustained throughout the lifespan (Kovács et al., 2019).

To date, no systematic studies are available comparing basal and stress-induced FOSB/ΔFOSB immunoreactivity in the rat central nervous system in multiple age groups.

Therefore, we aimed to detect the FOSB/ΔFOSB signal in the amygdala (MeA, BLA, and CeA), BNST (BNSTdl, BNSTov, BNSTdm, BNSTv, and BNSTfu), habenular nuclei (LHb, MHb), PVN, EWcp, and DR, in eight control age groups, as well as in ARS- and CVMS-exposed rats of the same age. To test whether the age-related changes were specific to stress-recruited centers, we also evaluated the 4th layer of the primary somatosensory cortex barrel field (S1).

We hypothesized an increased FOSB/ΔFOSB immunosignal upon ARS and CVMS, and that the magnitude of activation will be affected by age. We also thought that age-related dynamics of ARS and CVMS-induced FOSB/ΔFOSB immunoreactivity will show brain-area-specific dynamics.

## Materials and Methods

### Animals and Stress Procedures

**FIGURE 1:**
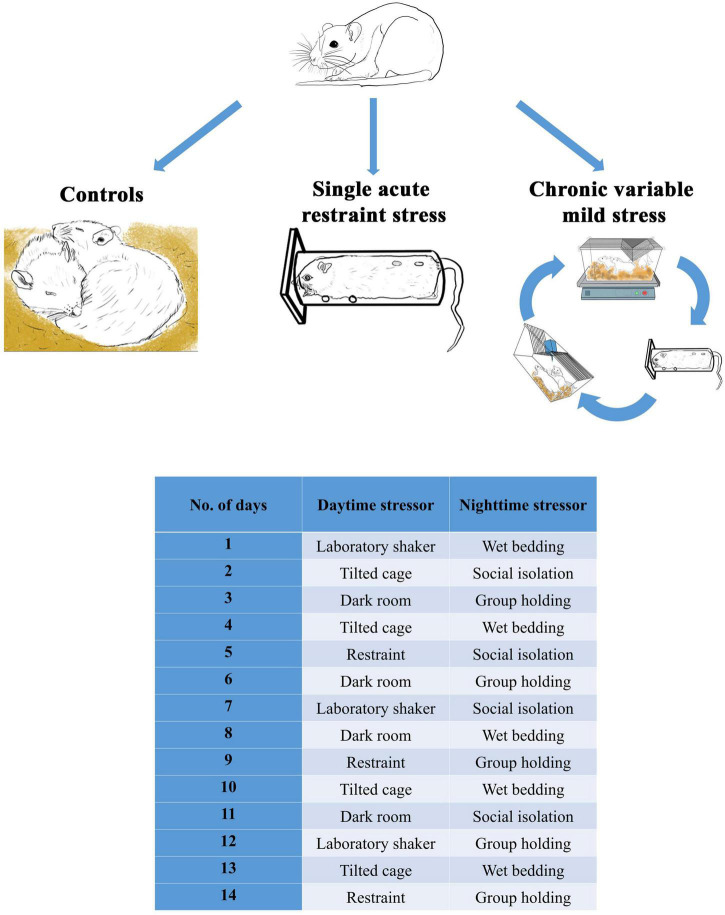
Experimental design and the 2-weeks-long chronic variable mild stress paradigm.

Tissue samples of one hundred and forty-four male Wistar rats were used. The experimental design was previously published (Kovács et al., 2019). Briefly, eight age groups were created: 1, 1.5, 2, 3, 6, 12, 18, and 24 M, and the effect of acute restraint (ARS) was tested in comparison with the control animals. A single 60-min acute restraint stress was applied as ARS as previously published (Kovács et al., 2018). In 2, 3, 6, 12, 18, and 24 M animals, the effect of CVMS exposure was examined in comparison with age-matched controls (Figure 1). The 2 weeks CVMS protocol includes a shorter daytime (1–3 h) stress exposure and an overnight (12 h) stressor (see full details in Figure 1 and Kovács et al., 2019). The daytime stressors include 1-h long restraint stress, 2 h of laboratory orbital shaker, 3 h of darkroom exposure, or 3 h of tilted (45–50*^o^*) cage (further detail see in Kovács et al., 2019). Animals in the ARS groups were sacrificed 2 h after the onset of restraint. CVMS animals were euthanized 24 h after the last stress exposure. To avoid a long-term storage-related bias affecting the results, the study was designed in a way that all rats were perfused within 4 weeks when they reached the required age.

The protocol was approved by the Animal Welfare Committee of Pécs University, the National Scientific Ethical Committee on Animal Experimentation in Hungary, and the National Food Chain Safety Office in Hungary (license numbers: BA02/2000-25/2011 and BA02/2000-24/2017).

### Sample Selection and Free-Floating Diaminobenzidine FOSB/ΔFOSB Immunolabeling

Due to the large sample size and complexity of the study, the FOSB/ΔFOSB labeling was performed in four “runs.” We randomized the samples in a way that each run contained 1–2 animals of all groups. The four runs were performed in 2 consecutive weeks. All the reagents were used from the same products/kits as detailed below. All efforts were done to keep all conditions considerably constant during the four runs. We used internal controls by putting the samples of five selected rats of different ages into all four runs. Then, we compared the cell count values of these five animals and ran an analysis of variance (ANOVA) to test potential differences. ANOVA did not detect any differences between these datasets. Finally, we also run an ANOVA analysis on all samples, where the staining run was defined as an extra categorical predictor. As no significant main effect of the runs was found, results obtained in the four runs were analyzed together.

Sections from our previously reported experiment (Kovács et al., 2019) were used. For long-term storage, the sections were kept in an anti-freeze solution at –20°C. Representative series of 30 μm coronal sections interspaced by 90 μm collected between the optic chiasm and pontomedullary boundary were stained. Sections were washed for 6 × 10 min in 0.1 M PBS and permeabilized with 0.5% Triton X-100 (Sigma Chemical). After incubation in 2% normal goat serum (NGS, Jackson Immunoresearch Europe Ltd., United Kingdom) in PBS for 30 min, sections were treated in polyclonal rabbit FOSB antiserum diluted to 1:16,000 (Abcam) in PBS for 16 h at room temperature. After PBS washes, sections were incubated in biotinylated goat anti-rabbit IgG diluted to 1:200 in PBS and 2% NGS (Vectastain ABC Elite kit, Vector Laboratories, Burlingame, CA, United States). After PBS washes, preparations were treated with an avidine-biotin complex solution in PBS (Vectastain ABC Elite kit). Then, the immunolabeling was visualized in Tris buffer containing 0.02% diaminobenzidine (D5637; Sigma Chemical, Zwijndrecht, Netherlands) and 0.03% H_2_O_2_. The reaction was controlled under a stereomicroscope and stopped by PBS after 8 min. The sections were mounted to gelatin-coated slides, air dried, cleared with xylene, and finally covered-slipped with DePex (Fluka, Heidelberg, Germany).

The specificity and sensitivity of the FOSB/ΔFOSB serum [Abcam (#EPR15905, RRID:AB_2721123)] were tested earlier in the rat by Bollinger et al. (2019). With the omission of the primary or secondary antiserum, their replacement with normal non-immune sera abolished the immunosignal in this experiment also. The pre-adsorption test for specificity was not applicable as the supplier does not provide the recombinant blocking peptide fragment and holds the exact peptide sequence as proprietary information. Comparison with an alternative FOSB antiserum (Abcam polyclonal mouse FOSB Cat No: AB11959) further supported the specificity (see also: Kovács et al., 2019). Western blot analysis also supports the specificity of this serum in the rat.^1^

### Microscopy and Digitalization

The coronal sections were selected based on the rat brain atlas of Paxinos and Watson (2007) as summarized in Table 1. For subdivisions of the BNST, we used the classifications of Dong et al. (2001) and Hammack et al. (2010).

**TABLE 1:** List of examined brain areas and their distance from Bregma in mm.

Brain area	Distance from Bregma	
	From (mm)	To (mm)
CeA	–1.80	–2.92
BLA	–2.04	–3.36
MeA	–2.16	–3.36
MHb	–3.12	–4.20
LHb	–3.12	–4.08
BNSTov	0.12	–0.24
BNSTdl	0.12	–0.36
BNSTdm	0.12	–0.36
BNSTv	0.12	–0.36
BNSTfu	0.12	–0.24
PVN	–1.56	–1.92
EWcp	–5.16	–6.72
DR	–6.84	–7.68
S1	–1.56	–2.64

*Central (CeA), basolateral (BLA), and medial (MeA) nuclei of the amygdala; oval (BNSTov), dorsolateral (BNSTdl), dorsomedial (BNSTdm), vental (BNSTv), and fusiform (BNSTfu) divisions of the bed nucleus of the stria terminalis (BNST); PVN, paraventricular nucleus of hypothalamus, medial- (MHb) and lateral- (LHb) habenula; EWcp, centrally-projecting Edinger-Westphal nucleus; DR, dorsal raphe nucleus; S1, primary somatosensory cortex, barrel field.*

**FIGURE 2:**
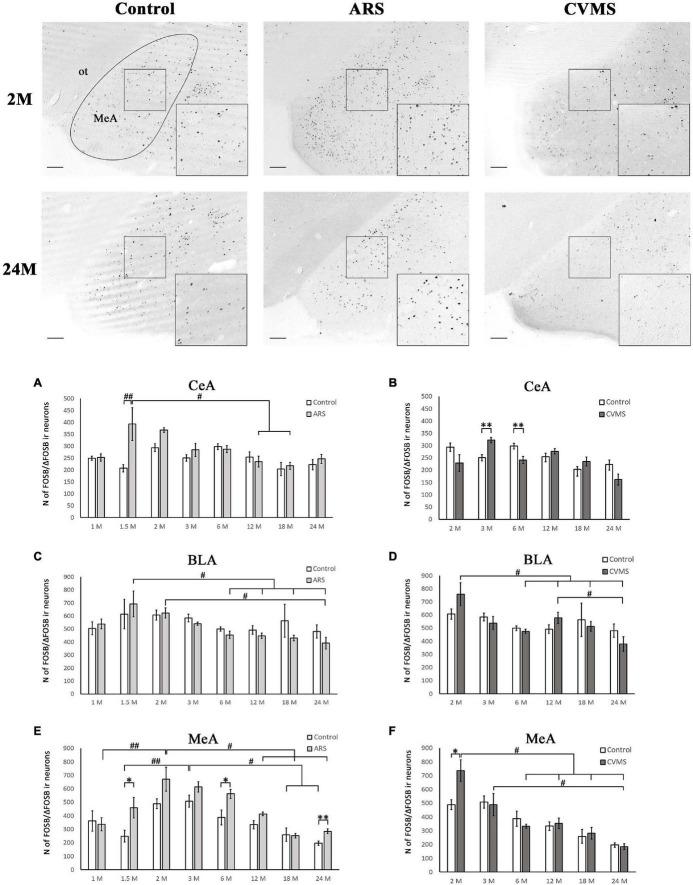
Age-dependent FOSB/ΔFOSB immunoreactivity (IR) in the amygdala. Representative images of 2- and 24-month-old (M) control, acute restraint stress (ARS) and chronic variable mild stress (CVMS)-exposed rats in the medial amygdala (MeA) at –3.24 mm to the Bregma. The numbers of FOSB/ΔFOSB IR nuclei were compared among eight age groups as demonstrated in panels **(A,B)** for the MeA, in panels **(C,D)** for central nucleus of amygdala (CeA) and in panels **(E,F)** for basolateral nucleus of amygdala (BLA). Open bars represent the control groups; light gray columns refer to ARS-exposed rats; the dark gray columns indicate the CVMS-exposed animals (*N* = 4–7). M, month-old; ot, optic tract. ^#^*p* < 0.05, ^##^*p* < 0.01 according to Tukey’s *post-hoc* test. *p < 0.05, ^**^p < 0.01 according to *t*-tests. Scale bar: 100 μm.

**FIGURE 3:**
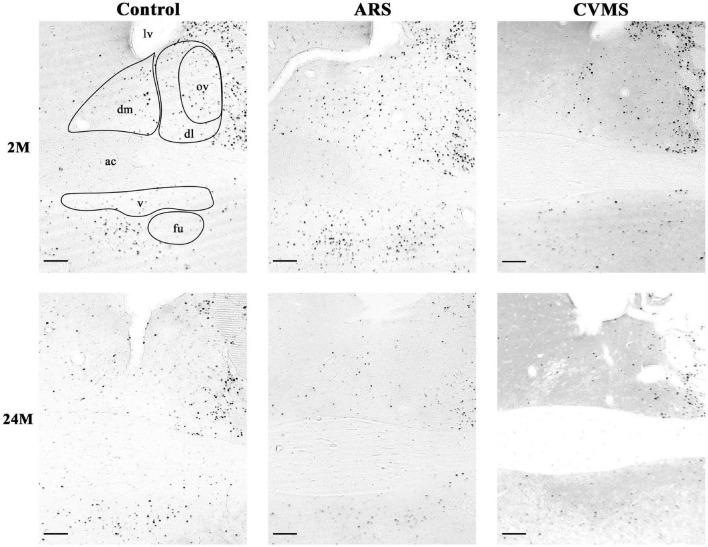
Representative photos of age-dependent FOSB/ΔFOSB immunoreactivity in the bed nucleus of the stria terminalis (BNST) in acute restraint stress (ARS)-exposed and chronic variable mild stress (CVMS)-subjected rats at 2-months-old (M) and at 24 M at –0.12 mm to the Bregma. dm, dorsomedial division of BNST; dl, dorsolateral division of BNST; ov, oval division of BNST; v, ventral division of BNST; fu, fusiform division of BNST; ac, anterior commissure; lv, lateral ventricle. Scale bar: 100 μm.

**FIGURE 4:**
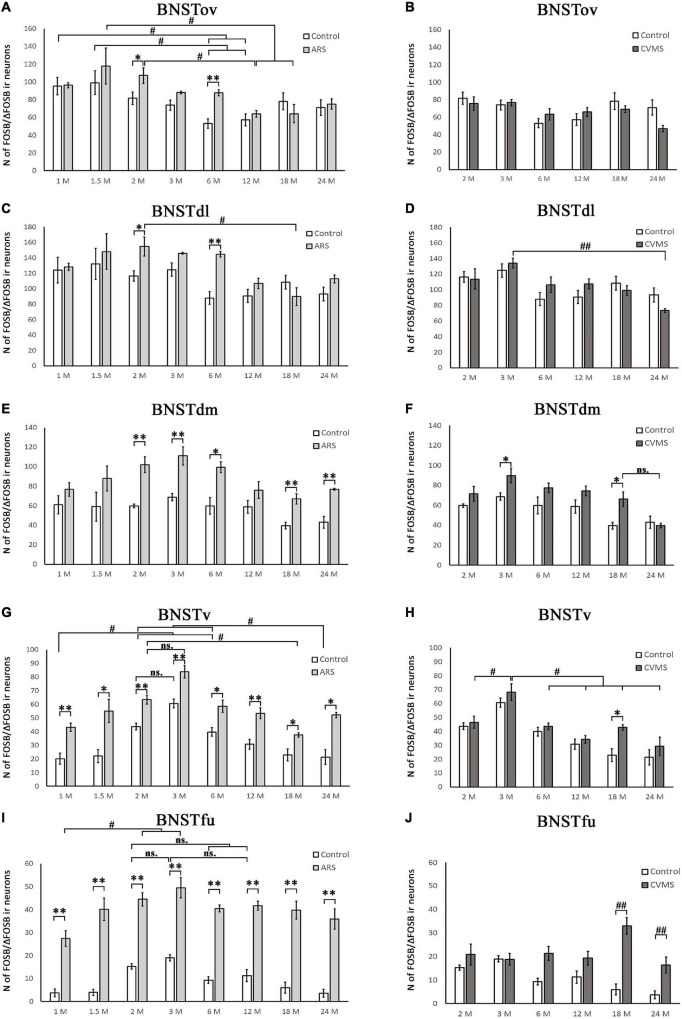
Diagrams illustrating age-dependent FOSB/ΔFOSB immunoreactivity (IR) in the bed nucleus of the stria terminalis (BNST). The number of FOSB/ΔFOSB IR nuclei was compared among eight age groups in two models. Control vs. acute restraint stress (ARS) and control vs. chronic variable mild stress (CVMS) comparisons are shown in histograms **(A,B)** for the oval (BNSTov); **(C,D)** for dorsolateral (BNSTdl); **(E,F)** for dorsomedial (BNSTdm); **(G,H)** for ventral (BNSTv), and **(I,J)** for the fusiform (BNSTfu) subdivisions of the BNST. Open bars represent the control groups; light gray columns refer to ARS-exposed rats; dark gray columns indicate the CVMS-exposed animals (*N* = 4–7). ^#^*p* < 0.05, ^##^*p* < 0.01 (ns, insignificant), according to Tukey’s *post-hoc* test. *p < 0.05, ^**^p < 0.01 according to *t*-tests. Representative images are shown in Figure 3.

**FIGURE 5:**
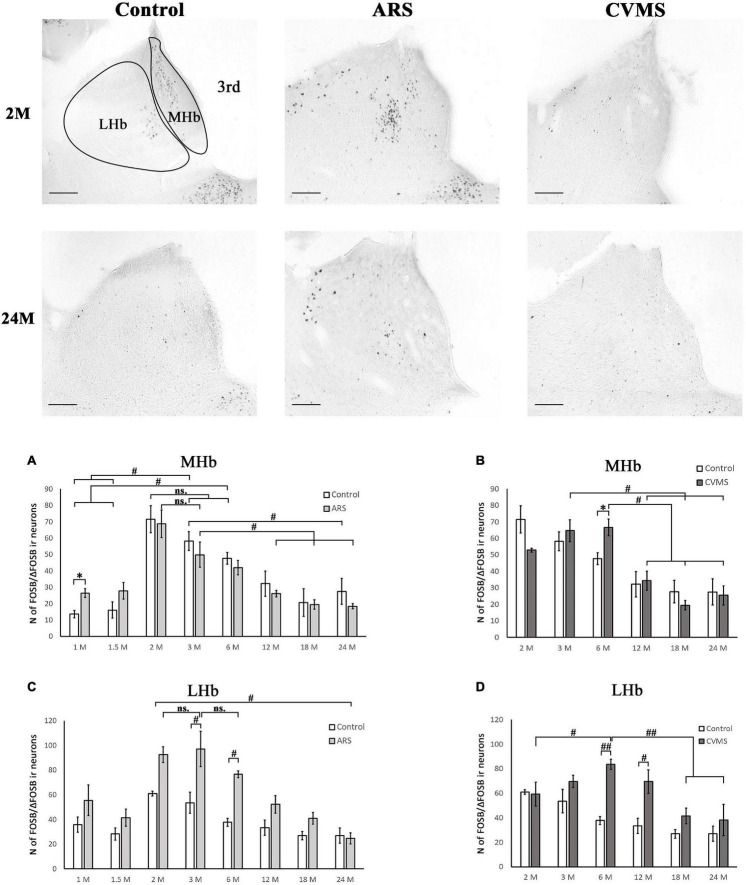
Age-dependent FOSB/ΔFOSB immunoreactivity (IR) in the medial (MHb) and lateral (LHb) habenula at –3.48 mm to the Bregma. Representative images of 2 and 24-month-old (M) control, acute restraint stress (ARS), and chronic variable mild stress (CVMS)-exposed rats. The number of FOSB/ΔFOSB IR nuclei was compared among eight age groups in histograms **(A,B)** for MHb and **(C,D)** for LHb. Open bars represent the control groups; light gray columns indicate ARS-exposed rats and the dark gray columns show CVMS-exposed rats (*N* = 4–7). ^#^*p* < 0.05, ^##^*p* < 0.01 (ns, not significant), according to Tukey’s *post-hoc* test. *p < 0.05, ^**^p < 0.01 according to *t*-tests. 3rd, third ventricle. Scale bar: 100 μm.

**FIGURE 6:**
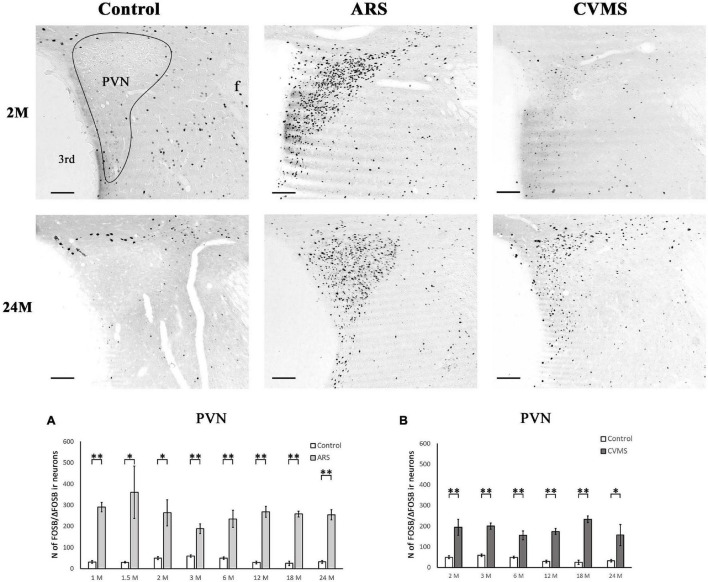
Age-dependent FOSB/ΔFOSB immunoreactivity (IR) in the paraventricular nucleus of the hypothalamus (PVN). Representative images of 2 and 24-month-old (M) control, acute restraint stress (ARS), and chronic variable mild stress (CVMS)-exposed rats at –1.72 mm to the Bregma. The number of FOSB/ΔFOSB IR nuclei was compared among eight age groups in histogram **(A)** for ARS vs. controls and **(B)** for CVMS vs. controls in the PVN. The ARS resulted in a significantly elevated FOSB/ΔFOSB in all age groups. The CVMS exposure resulted in FOSB/ΔFOSB increase in all age groups also. Open bars represent the control groups; light gray columns refer to ARS-exposed rats and the dark gray columns depict CVMS-exposed rats (*N* = 4–7). *p < 0.05, ^**^p < 0.01 according to *t*-tests. 3rd, third ventricle; f, fornix. Scale bar: 100 μm.

**FIGURE 7:**
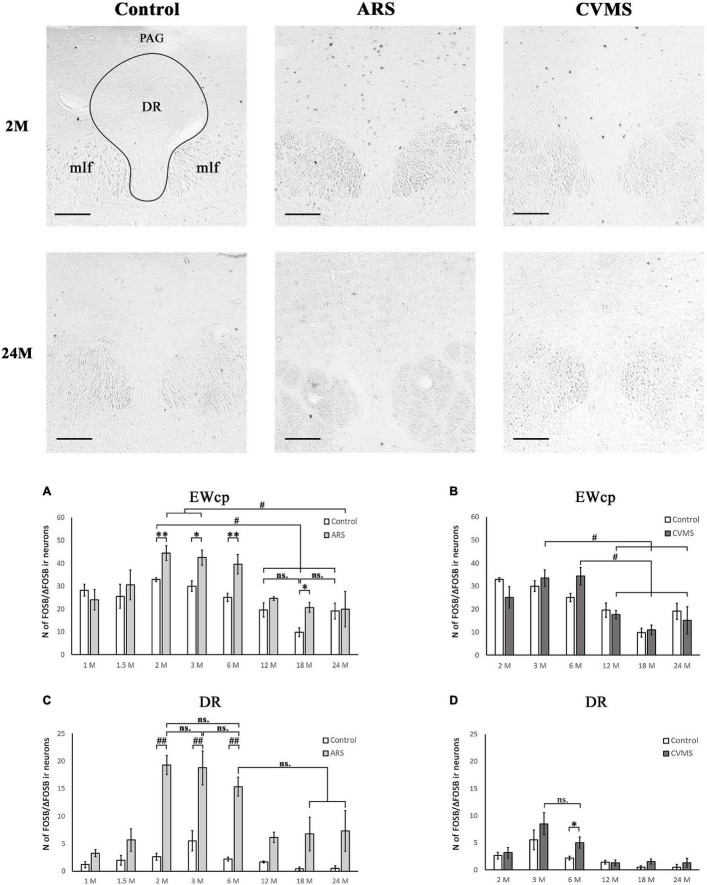
Age-dependent FOSB/ΔFOSB immunoreactivity (IR) in dorsal raphe (DR) and the centrally-projecting Edinger-Westphal (EWcp) nuclei. Representative images of DR in 2- and 24-month-old (M) control, acute restraint stress (ARS)- and chronic variable mild stress (CVMS)-exposed rats at –7.80 mm to the Bregma. The number of FOSB/ΔFOSB IR nuclei was compared among eight age groups in histogram **(A,B)** for DR and **(C,D)** for the EWcp. Open bars represent the control groups; light gray columns refer to ARS-exposed rats; the dark gray columns indicate the CVMS-exposed animals (*N* = 4–7). ^#^*p* < 0.05, ^##^*p* < 0.01 (ns, not significant) according to Tukey’s *post-hoc* test. *p < 0.05, ^**^p < 0.01 according to *t*-tests. mlf, medial longitudinal fasciculus; PAG, periaqueductal gray Scale bar: 100 μm.

**FIGURE 8:**
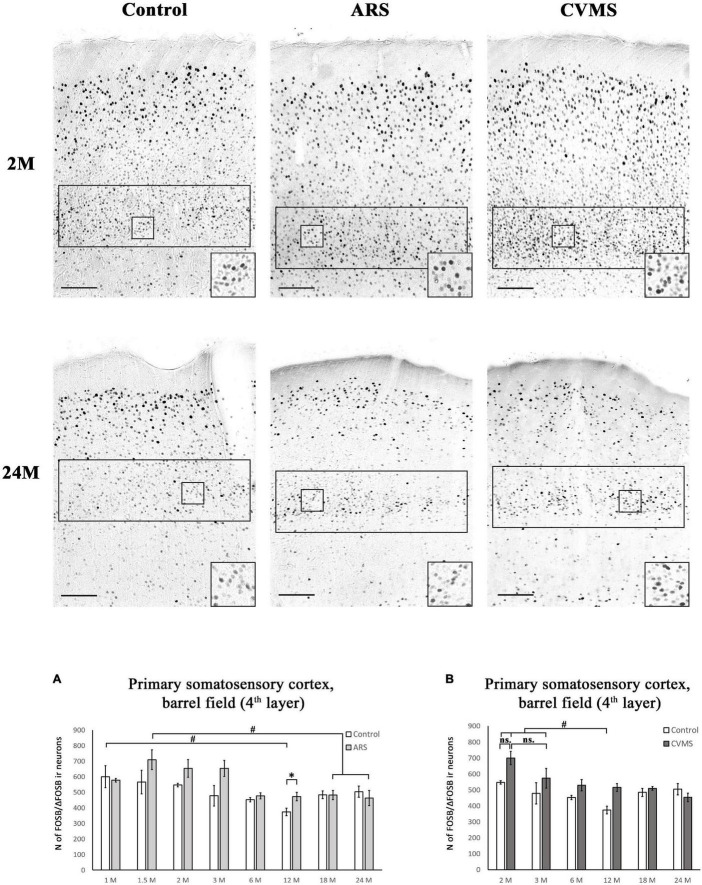
Age-dependent FOSB/ΔFOSB immunoreactivity (IR) in the primary somatosensory cortex, barrel field (S1). We examined the cell density of the 4th cortical layer at –3.12 mm to the Bregma. The images represent the S1 in 2- and 24-month-old (M) control, acute restraint stress (ARS)-, and chronic variable mild stress (CVMS)-exposed rats. The number of FOSB/ΔFOSB IR nuclei was compared among eight or six age groups in histogram **(A)** for ARS model, and **(B)** for the CVMS model. Open bars represent the control groups; light gray columns refer to ARS-exposed rats; the dark gray columns indicate the CVMS-exposed animals (*N* = 4–7). ^#^*p* < 0.05, ^##^*p* < 0.01, (ns, insignificant) according to Tukey’s *post-hoc* test. *p < 0.05, ^**^p < 0.01 according to *t*-tests. Scale bar: 100 μm.

A neurohistologist colleague, not informed about the identity of animals and groups, digitalized the sections using a Nikon Microphot FXA microscope with a real-time digital camera (Nikon, Tokyo Japan). Cell counts were determined with ImageJ manual cell counter mode. One brain area was analyzed by one person. Two colleagues supervised the cell counts by choosing pictures randomly and only confirmed data were used for the statistical assessment. At least five non-edited representative photos per brain area and animal were selected for morphometry. Cell counts of the five images of the respective brain region were averaged and this value represented one brain area of an animal. *N* = 4–7 animals per experimental group were included in the statistics. For publication purposes, selected representative digital images were grayscaled, contrasted, cropped, and edited into image montages (Figures 2–8) using Adobe Photoshop software.

### Statistical Analysis

All data were presented as the mean of the group with error bars representing the standard error of the mean. The normality of data distribution was tested by Shapiro and Wilk test (Shapiro and Wilk, 1965). The homogeneity of variances was tested by Hartley’s chi-square test (Snedecor and Cochran, 1989). Square root or natural logarithmic transformations were used to obtain the normal distribution of cell count data of PVN, CeA, and DR.

For statistical analysis, we used two-way ANOVA to test the main effect of stress and age, as well as their interaction. Tukey’s *post-hoc* test was applied to verify the differences between pairs of groups. In some cases, the two-way ANOVA did not detect a significant effect of interaction significantly. Here we tested the effects of age by one-way ANOVAs on controls, ARS, and CVMS groups, separately. In these cases, to test the effect of stress (ARS, CVMS) we used Student’s *t*-tests to compare age-matched pairs of groups (control vs. ARS and control vs. CVMS, respectively). To find an association between age and cell counts, we use Spearman’s rank correlation. The statistical difference was considered significant if alpha was lower than 5%. For all statistical analysis, we used Statistica 8.0. software (StatSoft, Tulsa, OK, United States).

## Results

### Results of the Acute Restraint Stress Model

#### Extended Amygdala

##### Central Nucleus of the Amygdala

The ANOVA found the main effect of age [*F*_(7_, _94)_ = 2.46; *p* < 0.05] and age × ARS interaction [*F*_(7_, _94)_ = 3.04; *p* < 0.01] on the count of FOSB/ΔFOSB immunoreactive cells significant (Table 2). Tukey’s *post-hoc* test detected a difference between control and ARS rats only in 1.5 M (*p* < 0.01) only (Figure 2A). This was the highest cell count that we found across ARS groups, and it was significantly higher than in 12 M (*p* < 0.05) or 18 M ARS (*p* < 0.05) animals. The Spearman analysis found a moderate negative correlation between age and cell counts only in ARS animals (ρ = –0.41; *p* < 0.05) (Table 3).

**TABLE 2:** Summary of the statistical results of the two-way ANOVAs in the acute restrain stress (ARS) model.

Brain area	Two-way ANOVA, main effects					
	Age		ARS		Age × ARS interaction	
	*F*-value	*p*-value	*F*-value	*p*-value	*F*-value	*p*-value
CeA	**2.46**	**<0.05**	2.63	0.11	**3.04**	**<0.01**
BLA	**3.57**	**<0.005**	0.97	0.33	0.67	0.69
MeA	**13.53**	**<10^–6^**	**15.40**	**<5** **×** **10^–4^**	1.43	0.21
BNSTov	**6.24**	**<5** **×** **10^–5^**	**6.00**	**<0.05**	1.26	0.28
BNSTdl	**4.57**	**<5** **×** **10^–4^**	**11.05**	**<0.005**	1.71	0.12
BNSTdm	**3.91**	**<0.005**	**56.85**	**<10^–6^**	0.92	0.50
BNSTv	**17.65**	**<10^–6^**	**105.8**	**<10^–6^**	0.83	0.56
BNSTfu	**8.25**	**<10^–5^**	**444.8**	**<10^–6^**	0.78	0.60
MHb	**19.64**	**<10^–6^**	0.12	0.73	1.04	0.41
LHb	**14.28**	**<10^–6^**	**36.14**	**<10^–6^**	**2.23**	**<0.05**
PVN	0.72	0.66	**166.9**	**<10^–6^**	1.48	0.19
EWcp	**8.23**	**<10^–6^**	**12.71**	**<0.001**	1.40	0.22
DR	**9.61**	**<10^–6^**	**87.44**	**<10^–6^**	**4.96**	**<5** **×** **10^–4^**
S1	**6.051**	**<10^–5^**	**7.38**	**<0.01**	1.68	0.13

*Bold letters indicate the significant main effects. Central (CeA), basolateral (BLA), and medial (MeA) nuclei of the amygdala; oval (BNSTov) dorsolateral (BNSTdl), dorsomedial (BNSTdm), ventral (BNSTv), fusiform (BNSTfu) divisions of the bed nucleus of the stria terminalis (BNST); medial- (MHb) and lateral- (LHb) habenula; PVN, paraventricular nucleus of hypothalamus; EWcp, centrally-projecting Edinger-Westphal nucleus; DR, dorsal raphe nucleus; S1, primary somatosensory cortex, barrel field.*

**TABLE 3:** Summary of Spearman’s correlation analyses.

Brain area	Control animals		ARS animals		CVMS animals	
	Spearman’s ρ	*p*	Spearman’s ρ	*p*	Spearman’s ρ	*p*
CeA	–0.12	0.47	**–0.41**	**<0.05**	**–0.54**	**<0.001**
BLA	–0.24	0.12	**–0.62**	**<5** **×** **10^–5^**	**–0.42**	**<0.05**
MeA	**–0.36**	**<0.05**	–0.29	0.07	**–0.70**	**<10^–5^**
BNSTov	**–0.41**	**<0.01**	**–0.68**	**<5** **×** **10^–5^**	**–0.48**	**<0.01**
BNSTdl	**–0.41**	**<0.01**	**–0.42**	**<0.05**	**–0.55**	**<0.005**
BNSTdm	**–0.38**	**<0.05**	–0.18	0.27	**–0.52**	**<0.005**
BNSTv	–0.10	0.52	–0.04	0.80	**–0.64**	**<5** **×** **10^–4^**
BNSTfu	–0.15	0.35	0.19	0.27	0.10	0.61
MHb	0.12	0.43	**–0.41**	**<0.05**	**–0.58**	**<0.001**
LHb	–0.26	0.09	–0.31	0.06	–0.32	0.08
PVN	–0.09	0.54	–0.08	0.62	–0.04	0.83
EWcp	**–0.55**	**<5** **×** **10^–4^**	–0.28	0.09	**–0.54**	**<0.001**
DR	**–0.32**	**<0.05**	–0.05	0.75	**–0.54**	**<0.001**
S1	**–0.34**	**<0.05**	**–0.63**	**<5** **×** **10^–5^**	**–0.54**	**<0.005**

*Bold letters indicate the significant correlations coefficient (ρ) and their p-values. ARS, acute restraint stress; CVMS, chronic variable mild stress; central (CeA), basolateral (BLA), and medial (MeA) nuclei of the amygdala; oval (BNSTov), dorsolateral (BNSTdl), dorsomedial (BNSTdm), ventral (BNSTv), fusiform (BNSTfu) divisions of the bed nucleus of the stria terminalis (BNST); medial (MHb) and lateral (LHb) habenula; PVN, paraventricular nucleus of hypothalamus; EWcp, centrally-projecting Edinger-Westphal nucleus; DR, dorsal raphe nucleus; S1, primary somatosensory cortex, barrel field.*

##### Basolateral Nucleus of the Amygdala

In lack of interaction effects in two-way ANOVA, we tested the influence of age in control and ARS separately by one-way ANOVA (Table 4). We found a significant main effect of age only in ARS animals [*F*_(7_, _46)_ = 5.13; *p* < 0.001]. The FOSB/ΔFOSB cell number of 1.5 M ARS animals was higher than in 6, 12, 18, or 24 M ARS rats (Tukey’s *post-hoc* test: *p* < 0.05). The 2 M ARS rats also presented a higher FOSB/ΔFOSB cell count than 24 M ARS animals (*p* < 0.05). Correlation analysis also supported a strong negative correlation between age and FOSB/ΔFOSB cell counts in ARS animals (ρ = –0.62; *p* < 5 × 10^–5^) (Table 3). According to the *t*-tests, ARS exposure did not affect the number of FOSB/ΔFOSB when compared to age-matched controls (Table 5 and Figure 2C).

**TABLE 4:** Summary of one-way ANOVA statistical analysis on age in acute restrain stress (ARS) and in chronic variable mild stress (CVMS) models.

Brain area	One-way ANOVA on age, main effects					
	Control groups		ARS groups		CVMS groups	
	*F*-value	*p*-value	*F*-value	*p*-value	*F*-value	*p*-value
CeA	1.13	0.37	**4.50**	**<0.005**	2.46	0.06
BLA	0.73	0.64	**5.13**	**<0.001**	**5.19**	**<0.005**
MeA	**7.13**	**<10^–4^**	**7.25**	**<10^–4^**	**7.29**	**<5** **×** **10^–4^**
BNSTov	**3.56**	**<0.01**	**3.85**	**<0.005**	2.23	0.08
BNSTdl	**2.51**	**<0.05**	**3.49**	**<0.01**	**4.36**	**<0.01**
BNSTdm	1.89	0.1	**2.74**	**<0.05**	**7.25**	**<0.001**
BNSTv	**11.55**	**<10^–6^**	**7.27**	**<10^–5^**	**10.63**	**<10^–5^**
BNSTfu	**8.91**	**<10^–5^**	**3.10**	**<0.05**	1.71	0.17
MHb	**10.84**	**<10^–6^**	**11.03**	**<5** **×** **10^–6^**	**8.30**	**<5** **×** **10^–4^**
LHb	**5.43**	**<5** **×** **10^–4^**	**8.77**	**<10^–5^**	**5.94**	**<0.005**
PVN	1.03	0.43	1.01	0.45	1.15	0.37
EWcp	**5.70**	**<5** **×** **10^–4^**	**4.01**	**<0.005**	**6.52**	**<5** **×** **10^–4^**
DR	**3.22**	**<0.01**	**7.13**	**<5** **×** **10^–5^**	**6.92**	**<5** **×** **10^–4^**
S1	**2.49**	**<0.05**	**3.59**	**<0.01**	**4.12**	**<0.01**

*Bold letters indicate the significant main effects. Central (CeA), basolateral (BLA), and medial (MeA) nuclei of the amygdala; oval (BNSTov), dorsolateral (BNSTdl), dorsomedial (BNSTdm), ventral (BNSTv), and fusiform (BNSTfu) divisions of the bed nucleus of the stria terminalis (BNST); medial (MHb) and lateral (LHb) habenula; PVN, paraventricular nucleus of hypothalamus; EWcp, centrally-projecting Edinger-Westphal nucleus; DR, dorsal raphe nucleus; S1, primary somatosensory cortex, barrel field.*

**TABLE 5:** Summary of the *p*-values of *t*-tests in the ARS model.

Brain area	Age group of animals							
	1 M	1.5 M	2 M	3 M	6 M	12 M	18 M	24 M
CeA	0.27	**<0.05**	**<0.003**	0.64	0.57	0.54	0.68	0.49
BLA	0.58	0.61	0.78	0.2	0.21	0.30	0.38	0.18
MeA	0.78	**<0.05**	0.08	0.11	**<0.05**	0.06	0.91	**<0.01**
BNSTov	0.92	0.51	**<0.05**	0.06	**<0.01**	0.42	0.36	0.75
BNSTdl	0.79	0.64	**<0.05**	0.06	**<0.001**	0.17	0.24	0.14
BNSTdm	0.18	0.20	**<5** **×** **10^–4^**	**<0.005**	**<0.01**	0.14	**<0.005**	**<0.01**
BNSTv	**<0.001**	**<0.05**	**<0.001**	**<0.01**	**<0.05**	**<0.005**	**<0.05**	**<0.01**
BNSTfu	**<5** **×** **10^–4^**	**<0.005**	**<5** **×** **10^–6^**	**<0.005**	**<5** **×** **10^–6^**	**<5** **×** **10^–6^**	**<5** **×** **10^–4^**	**<0.001**
MHb	**<0.01**	0.13	0.82	0.41	0.35	0.50	0.88	0.33
LHb	0.17	0.16	**<0.001**	**<0.05**	**<5** **×** **10^–5^**	0.08	0.07	0.78
PVN	**<5** **×** **10^–5^**	**<0.05**	**<0.05**	**<0.001**	**<0.005**	**<5** **×** **10^–6^**	**<5** **×** **10^–6^**	**<5** **×** **10^–5^**
EWcp	0.44	0.57	**<0.005**	**<0.05**	**<0.01**	0.19	**<0.05**	0.91
DR	**<0.05**	0.11	**<5** **×** **10^–6^**	**<0.01**	**<5** **×** **10^–5^**	**<0.001**	**<0.05**	0.09
S1	0.77	0.20	0.07	0.06	0.32	**<0.05**	0.96	0.5

*Comparisons of control vs. acute restrain stress (ARS) in all age groups. Bold letters indicate the significant differences. Central (CeA), basolateral (BLA), and medial (MeA) nuclei of the amygdala; oval (BNSTov), dorsolateral (BNSTdl), dorsomedial (BNSTdm), ventral (BNSTv), fusiform (BNSTfu) divisions of the bed nucleus of the stria terminalis (BNST); medial- (MHb) and lateral- (LHb) habenula, PVN, paraventricular nucleus of hypothalamus; EWcp, centrally-projecting Edinger-Westphal nucleus; DR, dorsal raphe nucleus; S1, primary somatosensory cortex, barrel field.*

##### Medial Nucleus of the Amygdala

The one-way ANOVA found the main effect of age in both controls [*F*_(7_, _48)_ = 7.13; *p* < 10^–4^] and ARS animals [*F*_(7_, _46)_ = 7.25; *p* < 5 × 10^–4^] noteworthy (Table 4). In controls, Tukey’s *post-hoc* tests revealed that the cell number peaked in 3 M animals, which was higher than in 1.5 M (*p* < 0.01), 18 M (*p* < 0.05), or 24 M controls (*p* < 5 × 10^–4^). In ARS groups, the maximal FOSB/FOSBs cell number was found in the 2 M animals. This was considerably higher than in 1 M (*p* < 0.001), 12 M (*p* < 0.05), 18 M (*p* < 5 × 10^–4^), or 24 M (*p* < 5 × 10^–4^) ARS animals. The 3 M ARS animals also exhibited higher cell count than 1 M (*p* < 0.005), 18 M (*p* < 0.005), or 24 M (*p* < 0.05) groups. The Spearman analysis found a weak negative correlation between age and FOSB/ΔFOSB cell counts in control animals (ρ = –0.36; *p* < 0.05) (Table 3). The *t*-tests confirmed the effect of ARS on FOSB/ΔFOSB cell counts in 1.5, 6, and 24 M (Table 5 and Figure 2E).

##### Oval Division of the Bed Nucleus of the Stria Terminalis

The one-way ANOVA found the effect of age significant in both controls [*F*_(7_, _48)_ = 3.56; *p* < 0.01] and ARS animals [*F*_(7_, _46)_ = 3.85; *p* < 0.005] (Table 4). In control animals, the 1 and 1.5 M groups had the highest cell counts and both groups exhibited higher cell counts than 6 or 12 M controls (*p* < 0.05). In ARS animals, the 1.5 M group had the top cell count and it was high compared to 12 M (*p* < 0.01) and 18 M (*p* < 0.05) rats. Similarly, 2 M ARS animals also differed from 12 M (*p* < 0.05) rats. A negative correlation was found between age and FOSB/ΔFOSB cell numbers both in control (ρ = –0.41; *p* < 0.01) and ARS groups (ρ = –0.68; *p* < 5 × 10^–5^) (Table 3). We also used *t*-tests to verify the effect of stress. ARS increased the FOSB/ΔFOSB cell counts only in the 2 and 6 M animals (Table 5 and Figures 3, 4A).

##### Dorsolateral Division of the Bed Nucleus of the Stria Terminalis

The one-way ANOVA confirmed the main effect of age both in control [*F*_(7_, _48)_ = 2.51; *p* < 0.05] and ARS-exposed animals [*F*_(7_, _46)_ = 3.49; *p* < 0.01] (Table 4). Tukey’s *post-hoc* test confirmed the age-related decline in FOSB/ΔFOSB ARS response as 18 M animals showed a lower signal than 2 M ARS rats (*p* < 0.05). Moderate negative correlations were detected between age and FOSB/ΔFOSB cell numbers in both controls (ρ = –0.41; *p* < 0.01) and ARS (ρ = –0.42; *p* < 0.05) groups (Table 3). The *t*-tests confirmed the effect of stress in 2 and 6 M animals (Table 5 and Figures 3, 4C).

##### Dorsomedial Division of the Bed Nucleus of the Stria Terminalis

The main effects of age were significant only in ARS animals [*F*_(7_, _46)_ = 2.74; *p* < 0.05] (Table 4), however, Tukey’s *post-hoc* test did not find any difference across ARS groups.

Only control animals showed a weak negative correlation between age and cell count according to the Spearman test (ρ = –0.38; *p* < 0.05) (Table 3). The comparisons of control and age-matched ARS animals by *t*-tests verified the effect of stress in five age groups: 2, 3, 6, 18, and 24 M (Table 5 and Figures 3, 4E).

##### Ventral Division of the Bed Nucleus of the Stria Terminalis

The one-way ANOVA proved the main effect of age in control animals [*F*_(7_, _48)_ = 11.55; *p* < 10^–6^] (Table 4). The highest cell count was detected in the 3 M controls, which was considerably greater than in 1 M (*p* < 5 × 10^–4^), 1.5 M (*p* < 5 × 10^–4^), 6 M (*p* < 0.05), 12 M (*p* < 5 × 10^–4^), 18 M (*p* < 5 × 10^–4^), and 24 M (*p* < 5 × 10^–4^). The lowest cell counts were observed in the youngest (1 M) and oldest (24 M) control animals. Both exhibited fewer cell counts than we observed in the 2 M (*p* < 0.005) and 6 M (*p* < 0.05) controls.

The ANOVA detected a significant main effect of age also in ARS-exposed animals [*F*_(7_, _46)_ = 7.27; *p* < 10^–5^] on FOSB/ΔFOSB cell count. The highest ARS cell count was detected in 3 M animals, which was higher compared to 1 M (*p* < 5 × 10^–4^), 1.5 M (*p* < 0.05), 3 M (*p* < 0.005), 6 M (*p* < 0.05), 12 M (*p* < 0.005), 18 M (*p* < 0.05), and 24 M (*p* < 0.01) groups. The Spearman test did not find any significant relationship between age and FOSB/ΔFOSB cell counts in the BNSTv (Table 3). ARS increased the FOSB/ΔFOSB cell counts in all age groups as supported by *t*-tests (Table 5 and Figures 3, 4G).

##### Fusiform Division of the Bed Nucleus of the Stria Terminalis

The one-way ANOVA supported the main effects of age both in controls [*F*_(7_, _48)_ = 8.91; *p* < 10^–5^] and ARS-exposed rats [*F*_(7_, _46)_ = 3.10; *p* < 0.05] (Table 4). In controls, Tukey’s *post-hoc* test revealed that 2 M animals exhibited higher cell numbers than detected in 1 M (*p* < 0.005), 1.5 M (*p* < 0.05), and 18 M (*p* < 0.05), and 24 M (*p* < 0.001) rats. The 3 M controls also had larger cell counts than 1 M (*p* < 5 × 10^–4^), 1.5 M (*p* < 0.005), 6 M (*p* < 0.05), 18 M (*p* < 0.005), and 24 M (*p* < 5 × 10^–4^) rats.

In ARS animals, the highest FOSB/ΔFOSB cell counts were detected in 2 and 3 M. Tukey’s tests showed that 1 M ARS animals had lower FOSB/ΔFOSB cell counts than 2 M (*p* < 0.05) and 3 M (*p* < 0.01) rats. The Spearman test did not find any linear correlations between age and cell numbers (Table 3). The effect of ARS was proven in all age groups by *t*-test (Table 5 and Figures 3, 4I).

#### Habenular Nuclei

##### Medial Habenular Nucleus

The one-way ANOVA proved the main effect of age in controls [*F*_(7_, _78)_ = 10.85; *p* < 10^–6^] and in ARS animals [*F*_(7_, _78)_ = 11.03; *p* < 10^–6^] (Table 4). Tukey’s *post-hoc* test confirmed that the FOSB/ΔFOSB neuron number was higher in 2 M control rats than in 1 M (*p* < 5 × 10^–4^), 1.5 M (5 × 10^–4^), 12 M (*p* < 0.005), 18 M (*p* < 0.005), and 24 M (*p* < 5 × 10^–4^). The cell count of 3 M controls also differed from 1 M (*p* < 0.001), 1.5 M (*p* < 0.005), and 24 M rats (*p* < 0.05).

The analysis proved that the 2 M ARS group had larger FOSB/ΔFOSB cell counts than detected in 1 M (*p* < 5 × 10^–4^), 1.5 M (5 × 10^–4^), 6 M (*p* < 0.05), 12 M (*p* < 5 × 10^–4^), 18 M (*p* < 5 × 10^–4^), and 24 M (*p* < 5 × 10^–4^) ARS rats. The 3 M ARS-exposed animals’ cell count was higher than in 12 M (*p* < 0.05), 18 M (*p* < 0.05), and 24 M (*p* < 0.005) ARS groups.

The Spearman analysis verified a moderate negative correlation between age and FOSB/ΔFOSB neuron number in ARS rats (ρ = –0.41; *p* < 0.05) (Table 3). The *t*-tests showed that only 1 M animals reacted to ARS (Table 5 and Figure 5A).

##### Lateral Habenular Nucleus

The two-way ANOVA revealed the main effect of age [*F*_(7_, _94)_ = 14.28; *p* < 10^–6^] and ARS [*F*_(1_, _94)_ = 36.14; *p* < 10^–6^], as well as their [*F*_(7_, _94)_ = 2.23; *p* < 0.05] significant interaction (Table 2 and Figure 5C). Tukey’s *post-hoc* test detected significant differences between control and ARS animals at 3 M (*p* < 0.05) and 6 M (*p* < 0.05) age groups. The highest FOSB/ΔFOSB immunoreactivity was detected in 3 M ARS animals, which was greater than in any other groups (*p* < 0.05), except for 2 M and 6 M ARS rats. The highest basal immunoreactivity was found in 2 M control animals, which was significantly higher than in 24 M controls. The Spearman analysis did not detect any age-associated linear correlations (Table 3).

#### Paraventricular Nucleus of the Hypothalamus

The one-way ANOVA did not find the main effect of age significant in controls and ARS groups (Table 4). Accordingly, the Spearman test did not detect any relationship between age and cell counts (Table 3). The *t*-tests revealed that the ARS exposure significantly increased FOSB/ΔFOSB in all age groups (Table 5 and Figure 6A).

#### Centrally-Projecting Edinger-Westphal Nucleus

The main effect of age tested by one-way ANOVA was significant both in control [*F*_(7_, _48)_ = 5.70; *p* < 5 × 10^–4^] and ARS [*F*_(7_, _46)_ = 4.01; *p* < 0.005] rats (Table 4). The lowest cell number in controls was found in the 18 M animals compared to 1 M (*p* < 0.01), 1.5 M (*p* < 0.05), 2 M (*p* < 5 × 10^–4^), 3 M (*p* < 0.005), and 6 M (*p* < 0.05) controls. The 2 M control group differed from both the 12 M (*p* < 0.05) and 24 M (*p* < 0.05) control animals.

In ARS, the 24 M group showed the lowest FOSB/ΔFOSB cell count, which differed significantly from the 2 M (*p* < 0.05) and 3 M (*p* < 0.05) ARS groups. The Spearman analysis proved a strong negative correlation between age and cell numbers only in control animals (ρ = –0.55; *p* < 5 × 10^–4^) (Table 3). ARS exposure increased FOSB/ΔFOSB immunoreactivity in 2, 3, 6, and 18 M groups (Table 5 and Figure 7A).

#### Dorsal Raphe Nucleus

The two-way ANOVA found the main effects of age [*F*_(7_, _94)_ = 9.61 *p* < 10^–6^], ARS [*F*_(1_, _94)_ = 87.44 *p* < 10^–6^] and their interaction [*F*_(7_, _94)_ = 4.96 *p* < 5 × 10^–4^] on DR FOSB/ΔFOSB immunoreactivity significantly (Table 2 and Figure 7C). ARS groups at age of 2, 3, and 6 months exhibited higher cell counts than their respective controls (*p* < 0.005). The highest values were detected in 2 and 3 M ARS animals and these values were significantly higher than in other ARS groups (except 6 M ARS rats) or control animals (*p* < 5 × 10^–5^), respectively. The 6 M ARS rats had higher cell counts than any of the control animals (*p* < 0.005) or 1 M (*p* < 0.005), 1.5 M (*p* < 0.05), and 12 M (*p* < 0.05) ARS groups. Age and FOSB/ΔFOSB cell counts showed a weak negative correlation in control animals (ρ = –0.32; *p* < 0.05) only (Table 3).

#### Primary Somatosensory Cortex, Barrel Field

The one-way ANOVA found a significant main effect of age in both controls [*F*_(7_, _48)_ = 2.49; *p* < 0.05] and ARS animals [*F*_(7_, _46)_ = 3.59; *p* < 0.01] (Table 4). In control animals, the highest cell count was detected in 1 M and that was somewhat higher than in the 12 M control group (*p* < 0.05). In ARS groups, 1.5 M animals had the highest cell number, and this value was greater than observed in 12 M and 24 M ARS groups (*p* < 0.05). The Spearman analysis supported a weak negative correlation between age and FOSB/ΔFOSB cell count in control animals (ρ = –0.34; *p* < 0.05) and found a stronger negative correlation in ARS animals (ρ = –0.63; *p* < 5 × 10^–5^) (Table 3). The effect of ARS was only significant in 12 M rats only (Table 5 and Figure 8A).

### Results of the Chronic Variable Mild Stress Model

#### Extended Amygdala Nuclei

##### Central Nucleus of the Amygdala

The one-way ANOVA did not reveal the main effect of age [*F*_(5_, _63)_ = 2.46; *p* = 0.06] (Table 4) significant. In contrast, the Spearman analysis supported a strong negative correlation between age and FOSB/ΔFOSB cell count in CVMS animals (ρ = –0.54; *p* < 0.001) (Table 3). The *t*-tests proved that CVMS affected FOSB/ΔFOSB cell counts in 3 and 6 M (Table 6 and Figure 2B).

**TABLE 6:** Summary of *p*-values in *t*-tests for stress effect in the CVMS model.

Brain area	Age group of animals					
	2 M	3 M	6 M	12 M	18 M	24 M
CeA	0.11	**<0.005**	**<0.05**	0.37	0.08	0.08
BLA	0.1	0.45	0.34	0.14	0.41	0.18
MeA	**<0.05**	0.83	0.33	0.70	0.73	0.66
MHb	0.16	0.48	**<0.05**	0.83	0.45	0.87
LHb	0.18	0.13	**<5** **×** **10^–6^**	**<0.05**	0.16	0.39
BNSTov	0.57	0.71	0.29	0.33	0.48	0.07
BNSTdl	0.19	0.41	0.21	0.14	0.48	0.17
BNSTdm	0.13	**<0.05**	0.12	0.08	**<0.05**	0.75
BNSTv	0.57	0.35	0.38	0.46	**<0.05**	0.45
BNSTfu	0.21	0.97	**<0.05**	0.06	**<0.005**	**<0.05**
PVN	**<0.01**	**<5** **×** **10^–5^**	**<0.005**	**<5** **×** **10^–6^**	**<5** **×** **10^–5^**	**<0.05**
EWcp	0.11	0.45	0.05	0.6	0.67	0.58
DR	0.63	0.31	**<0.05**	0.87	0.09	0.4
S1	**<0.005**	0.33	0.07	**<0.005**	0.38	0.29

*Comparisons of control vs. chronic variable mild stress (CVMS) groups in all age groups. Bold letters indicate the significant differences. Central (CeA), basolateral (BLA), and medial (MeA) nuclei of the amygdala; oval (BNSTov), dorsolateral (BNSTdl), dorsomedial (BNSTdm), ventral (BNSTv), fusiform (BNSTfu) divisions of the bed nucleus of the stria terminalis (BNST); medial- (MHb) and lateral- (LHb) habenula; PVN, paraventricular nucleus of hypothalamus; EWcp, centrally-projecting Edinger-Westphal nucleus; DR, dorsal raphe nucleus; S1, primary somatosensory cortex, barrel field.*

##### Basolateral Nucleus of the Amygdala

According to one-way ANOVA, the main effect of age on FOSB/ΔFOSB was significant [*F*_(5_, _39)_ = 5.19; *p* < 0.005] in CVMS animals (Table 4). Tukey’s *post-hoc* test found a significant difference between 2 and 6 M (*p* < 0.05) and 18 M (*p* < 0.05), as well as 24 M (*p* < 0.001) CVMS groups. The 12 and 24 M groups also differed (*p* < 0.05). In CVMS animals, a moderate negative correlation was found between age and FOSB/ΔFOSB cell counts (ρ = –0.42; *p* < 0.05) (Table 3). The t-tests did not find any differences between controls and age-matched CVMS groups (Table 6 and Figure 2D).

##### Medial Nucleus of the Amygdala

The one-way ANOVA revealed the main effects of age in CVMS animals are significant [*F*_(5_, _39_ = 7.29; *p* < 5 × 10^–4^; Table 4]. The highest FOSB/ΔFOSB cell count was detected in the 2 M CVMS group that differed from 6 M (*p* < 0.01), 12 M (*p* < 0.05), 18 M (*p* < 0.005), and 24 M (*p* < 5 × 10^–4^) CVMS groups significantly. The count of FOSB/ΔFOSB cells was higher in 3 M than in 24 M CVMS animals (*p* < 0.05). We found a strong negative correlation between age and FOSB/ΔFOSB neuron count in CVMS animals (ρ = –0.70; *p* < 10^–5^) (Table 3). According to the *t*-tests, CVMS affected the FOSB/ΔFOSB immunoreactivity only in 2 M rats (Table 6 and Figure 2F).

##### Oval Division of the Bed Nucleus of the Stria Terminalis

The one-way ANOVA did not find the main effect of age [*F*_(5_, _39)_ = 3.23 *p* = 0.08] that is significant on FOSB/ΔFOSB cell counts (Table 4). The Spearman correlation analysis revealed that age correlated negatively with FOSB/ΔFOSB cell counts in CVMS (ρ = –0.48; *p* < 0.005) animals (Table 3). The CVMS did not have any effect according to *t*-tests (Table 6 and Figures 3, 4B).

##### Dorsolateral Division of the Bed Nucleus of the Stria Terminalis

The one-way ANOVA supported the main effect of age [*F*_(5_, _39)_ = 4.36; *p* < 0.01] (Table 4). Tukey’s *post-hoc* test found a significant difference between 3 and 24 M CVMS animals (*p* < 0.005). The Spearman analysis showed that age and FOSB/ΔFOSB count negatively correlated in CVMS animals (ρ = –0.55; *p* < 0.005) (Table 3). According to *t*-tests, CVMS did not change FOSB/ΔFOSB immunoreactivity significantly (Table 6 and Figures 3, 4D).

##### Dorsomedial Division of the Bed Nucleus of the Stria Terminalis

In the CVMS animals, one-way ANOVA detected the main effects of age on FOSB/ΔFOSB significantly [*F*_(5_, _39)_ = 7.25; *p* < 0.001] (Table 4). *Post-hoc* tests found that the cell count in 24 M rats was lower than in 2 M (*p* < 0.05), 3 M (*p* < 0.001), 6 M (*p* < 0.005), and 12 M (*p* < 0.01) groups. We found a moderate negative correlation between age and cell counts in CVMS exposed rats (ρ = –0.52; *p* < 0.005) (Table 3). The *t*-tests found significant differences between control and CVMS rats in 3 and 18 M (Table 6 and Figures 3, 4F).

##### Ventral Division of the Bed Nucleus of the Stria Terminalis

The one-way ANOVA found that age [*F*_(5_, _39)_ = 10.63; *p* < 5 × 10^–5^] affected FOSB/ΔFOSB in the CVMS animals (Table 4). Tukey’s *post-hoc* test showed that the 3 M animals’ cell number was higher than in 2 M (*p* < 0.05), 6 M (*p* < 0.01), 12 M (*p* < 5 × 10^–4^), 18 M (*p* < 0.05), and 24 M (5 × 10^–4^) CVMS animals. In CVMS rats, the BNSTv FOSB/ΔFOSB cell count showed a strong negative correlation with age (ρ = –0.64; *p* < 0.005) (Table 3). CVMS elevated the FOSB/ΔFOSB immunoreactivity in 18 M (Table 6 and Figures 3, 4H).

##### Fusiform Nucleus of the Bed Nucleus of the Stria Terminalis

ANOVA detected that the main effects of age [*F*_(5_, _85)_ = 2.56; *p* < 0.05], CVMS [*F*_(1_, _85)_ = 49.70; *p* < 10^–6^] and their interaction [*F*_(5_, _85)_ = 4.83; *p* < 0.005] exerted a strong influence on BSNTfu FOSB/ΔFOSB cell counts (Table 7 and Figures 3, 4J). Tukey’s *post-hoc* tests found differences between 18 M (*p* < 5 × 10^–4^) and 24 M (*p* < 0.01) controls and age-matched CVMS animals. The CVMS groups did not differ from each other. Spearman’s analysis did not find a correlation between age and BSNTfu FOSB/ΔFOSB cell counts in CVMS exposed rats (ρ = 0.1; *p* = 0.61) (Table 3).

**TABLE 7:** Summary of the statistical results of the two-way ANOVAs in chronic variable mild stress (CVMS) model.

Brain area	Two-way ANOVA, main effects					
	Age		CVMS		Age × CVMS interaction	
	*F*-value	*p*-value	*F*-value	*p*-value	*F*-value	*p*-value
CeA	**2.51**	**<0.05**	0.06	0.80	1.13	0.35
BLA	**4.37**	**<0.01**	0.008	0.93	1.46	0.22
MeA	**15.33**	**<10^–6^**	0.27	0.60	1.03	0.41
BNSTov	**3.23**	**<0.05**	0.23	0.63	1.38	0.25
BNSTdl	**6.52**	**<10^–4^**	0.37	0.54	1.34	0.26
BNSTdm	**10.11**	**<10^–5^**	**20.02**	**<5** **×** **10^–5^**	1.33	0.27
BNSTv	**21.30**	**<10^–6^**	**9.25**	**<0.005**	0.98	0.44
BNSTfu	**2.56**	**<0.05**	**49.70**	**<10^–6^**	**4.83**	**<0.005**
MHb	**10.97**	**<10^–6^**	0.07	0.79	2.38	0.051
LHb	**7.21**	**<10^–4^**	**25.18**	**<10^–6^**	**4.89**	**<0.005**
PVN	0.70	0.62	**118.5**	**<10^–6^**	1.58	0.18
EWcp	**12.78**	**<10^–6^**	0.002	0.97	1.68	0.15
DR	**11.55**	**<10^–6^**	**5.70**	**<0.05**	0.83	0.53
S1	**7.01**	**<10^–4^**	**14.09**	**<5** **×** **10^–4^**	**2.71**	**<0.05**

*Bold letters indicate the significant main effects. Central (CeA), basolateral (BLA), and medial (MeA) nuclei of the amygdala; oval (BNSTov), dorsolateral (BNSTdl), dorsomedial (BNSTdm), ventral (BNSTv), fusiform (BNSTfu) divisions of the bed nucleus of the stria terminalis (BNST); medial- (MHb) and lateral- (LHb) habenula; PVN, paraventricular nucleus of hypothalamus; EWcp, centrally-projecting Edinger-Westphal nucleus; DR, dorsal raphe nucleus; S1, primary somatosensory cortex, barrel field.*

#### Habenular Nuclei

##### Medial Habenular Nucleus

The one-way ANOVA reported the main effect of age in the CVMS animals (Table 4) is significant [*F*_(5_, _39)_ = 8.30; *p* < 5 × 10^–4^]. According to Tukey’s *post-hoc* tests, the FOSB/ΔFOSB cell count of the 3 M CVMS rats differed from the 12 M (*p* < 0.05), 18 M (*p* < 0.005), and 24 M (*p* < 0.05) CVMS rats. The cell number of 6 M was higher than in 12 M (*p* < 0.05), 18 M (*p* < 0.005) and 24 M (*p* < 0.005) CVMS rats. The Spearman test detected a moderate negative correlation between MHb FOSB/ΔFOSB cell counts and age in CVMS animals (ρ = –0.58; *p* < 0.001) (Table 3). CVMS increased the cell count only in the 6 M group (*p* < 0.05) (Table 6 and Figure 5B).

##### Lateral Habenular Nucleus

The two-way ANOVA confirmed the main effects of age [*F*_(5_, _85)_ = 7.21; *p* < 10^–4^] and CVMS [*F*_(1_, _85)_ = 25.18; *p* < 10^–6^]. Moreover, the second-order effect of age × CVMS interaction on LHb FOSB/ΔFOSB cell count was recognized [*F*_(5_, _85)_ = 4.89; *p* < 0.005] (Table 7 and Figure 5D). Tukey’s test verified that the 6 M (*p* < 0.005) and 12 M (*p* < 0.05) CVMS animals presented higher cell counts compared to their respective controls. The 6 M CVMS animals had the highest FOSB/ΔFOSB cell count that differed significantly from 2 M (*p* < 0.05), 18 M (*p* < 0.001), and 24 M (*p* < 0.005) CVMS animals. The Spearman’s test did not find any correlation between age and FOSB/ΔFOSB content in CVMS animals (ρ = –0.32; *p* = 0.08) (Table 3).

#### Paraventricular Nucleus of the Hypothalamus

The one-way ANOVA did not find the main effect of age significant in CVMS animals (Table 4). Spearman’s correlation analysis did not detect any linear relationship between PVN FOSB/ΔFOSB cell count and age in CVMS animals (ρ = –*0.04*; *p* = 0.83) (Table 4). CVMS increased the cell count in all age groups as verified by *t*-tests (Table 6 and Figure 6B).

#### Centrally-Projecting Edinger-Westphal Nucleus

The one-way ANOVA proved the main effect of age [*F*_(5_, _39)_ = 6.51; *p* < 5 × 10^–4^] is significant (Table 4) in CVMS animals. The highest cell count was found in the 3 and 6 M CVMS animals. Both presented more active cells than 12 M (*p* < 0.05), 18 M (*p* < 0.005), or 24 M (*p* < 0.05) CVMS rats, according to the *post-hoc* tests. FOSB/ΔFOSB cell count decreased moderately with age in CVMS-exposed rats (ρ = –*0.54*; *p* < 0.001) (Table 3). The *t*-tests did not confirm any effect of CVMS on FOSB/ΔFOSB cell counts in the EWcp (Table 6 and Figure 7B).

#### Dorsal Raphe Nucleus

The one-way ANOVA detected the main effect of age [*F*_(5_, _39)_ = 6.92; *p* < 5 × 10^–4^] on DR FOSB/ΔFOSB cell count (Table 4) in CVMS groups. The cell count was higher in 3 M CVMS animals than in all other CVMS groups, except for 6 M animals. The FOSB/ΔFOSB cell count upon CVMS showed a moderate linear correlation with age (ρ = –*0.54*; *p* < 0.001) (Table 3). The CVMS effect on FOSB/ΔFOSB in the DR was confirmed in 6 M rats by the *t*-tests (Table 6 and Figure 7D).

#### Primary Somatosensory Cortex, Barrel Field

The two-way ANOVA found the main effects of age [*F*_(5_, _85)_ = 7.01; *p* < 10^–4^], CVMS [*F*_(1_, _85)_ = 14.09; *p* < 5 × 10^–4^] and their interaction [*F*_(5_, _85)_ = 2.71; *p* < 0.05] is significant (Table 7 and Figure 8B). Tukey’s *post-hoc* test did not confirm any CVMS-induced cell count rise at any age. The highest FOSB/ΔFOSB cell count was observed in 2 M among the controls, and this was greater than those in 12 M controls. The largest neuron number was observed in the 2 M CVMS animals, which was significantly higher than in any other groups, with exception of 2 M control and 3 M CVMS animals. Spearman analyses found a moderate negative correlation between age and FOSB/ΔFOSB cell count in CVMS animals (ρ = –*0.54*; *p* < 0.005) (Table 3).

## Discussion

### The Age-Related Dynamics of Basal FOSB/ΔFOSB Immunoreactivity Are Brain Area-Specific and Differ From That of FOS

The nuclei of the extended amygdala and S1 are characterized by high basal FOSB/ΔFOSB immunoreactivity (Herdegen et al., 1995; Perrotti et al., 2004; Kovács et al., 2019); the habenular nuclei (Zhang et al., 2018) and EWcp (Kormos et al., 2016) shows a moderate level; while the DR (Kormos et al., 2016; Farkas et al., 2017) and the PVN (Chocyk et al., 2006; Das et al., 2009; Kovács et al., 2019) display very low FOSB/ΔFOSB signal. The comparison of basal FOSB/ΔFOSB with the basal’s FOS immunoreactivity reveals lower FOS signal in the extended amygdala (Kellogg et al., 1998; Kovács et al., 2018), S1 (Kovács et al., 2018), and habenula (Chastrette et al., 1991; Sood et al., 2018).

The basal FOSB/ΔFOSB immunoreactivity in the CeA, BLA, BNSTdm, and PVN did not display any age-dependent changes. The cases of CeA, BLA, and BNSTdl are contrary to the age-dependent decline of basal’s FOS immunoreactivity (Kovács et al., 2018). The BNSTdl and S1 cell counts showed some age-dependent change according to the ANOVA, but the decline was not unequivocally proven by *post-hoc* tests. In the case of the S1, only the 12 M group differed significantly. These considerations suggest that the influence of age on FOSB/ΔFOSB in the BNSTdl and S1 might be very limited.

Five nuclei (MeA, BNSTv, BNSTfu, MHb, and LHb) exhibit an increasing basal FOSB/ΔFOSB signal from the puberty (1–1.5 M) on to late adolescence/early adulthood (2–3 M), and, thereafter, the immunosignal decreases to the senescence (18–24 M). These data may reflect neural maturation during the adolescence and early adult periods (1–2 months of age) reaching the maximal basal activity in young adults (3 months) (McCutcheon and Marinelli, 2009; Sengupta, 2013). Five nuclei (BNSTov, BNSTdl, BNSTdm, EWcp, and DR) did not exhibit these reversed U-shaped dynamics during the examined life period. Instead, they were characterized by a gradual decrease of FOSB/ΔFOSB immunosignal by aging.

#### The Acute Restraint Stress-Evoked FOSB/ΔFOSB Depends on Age in a Brain Area-Specific Manner

In line with earlier studies (Kang et al., 2021), the ARS exposure did not change the BLA FOSB/ΔFOSB, compared to controls. The FOSB/ΔFOSB’s induction was detected only at 1 M in the MHb, while older animals did not show any ARS-induced FOSB/ΔFOSB rise. As the ARS-evoked, FOS increase was shown here earlier (Sood et al., 2018) and it seems that MHb FOS and MHb FOSB/ΔFOSB are differentially regulated by ARS. The S1 did not denote any remarkable FOSB/ΔFOSB rise, which is strongly contradictory to its FOS response (Kovács et al., 2018), but it might be explained by the high basal expression and refers, again, to the notion that FOS and FOSB have different roles and dynamics.

In 11 nuclei (CeA, MeA, BNSTov, BNSTdl, BNSTdm, BNSTv, BNSTfu, LHb, PVN, DR, and EWcp), the FOSB/ΔFOSB increased after ARS exposure, but this phenomenon was highly age-dependent. The BNSTfu, BNSTv, and PVN reacted throughout the examined lifespan, which is in line with the unaltered magnitude of the HPA axis CORT response (Herman et al., 2001; Sterrenburg et al., 2011, 2012; Kovács et al., 2018). Eight nuclei (CeA, MeA, BNSTov, BNSTdl, BNSTdm LHb EWcp, and DR) displayed an FOSB/ΔFOSB response in specific age periods. In 2 M, we saw a significant rise in six nuclei (CeA, BNSTov, BNSTdl, BNSTdm, DR, and EWcp). Four nuclei (BNSTdm, LHb, EWcp, and DR) responded to ARS with increased FOSB/ΔFOSB immunoreactivity in 3 M, and five (MeA, BNSTov, BNSTdl, BNSTdm, LHb, and DR) nuclei in 6 M. Younger (1, 1.5 M) and older age groups (beyond 12 M) showed lower reactivity to ARS.

It seems that rats at younger age periods (2 and 3 M) exhibit higher susceptibility, which coincides with the critical period of increased vulnerability to psychopathologies (McCormick et al., 2010). Indeed, the magnitude of ARS-induced FOSB/ΔFOSB’s rise was the greatest at 1.5–3 M, which represents the late adolescence and early adulthood (Spear, 2000; McCutcheon and Marinelli, 2009; Sengupta, 2013). This period is characterized by increased sensitivity and responsivity of the brain (McCormick et al., 2010; Kovács et al., 2018, 2019), but we have fewer data about later adulthood. Here we saw that MeA, BNSTov, BNSTdl, BNSTdm, LHb, and DR maintain their responsivity.

#### The Acute Restraint Stress-Evoked FOSB/ΔFOSB Immunoreactivity Is a Function of Age

The comparison of the age-dependent FOSB/ΔFOSB dynamics in the examined brain areas revealed that five nuclei (MeA, BNSTv, MHb, LHb, and DR) showed relatively low FOSB/ΔFOSB immunoreactivity at 1 and 1.5 M that increased in 2-3 M, followed by a decrease in adulthood (12 M), early (18 M), and late senescence (24 M). Six other regions (CeA, BLA, BNSTov, BNSTdl, EWcp, and S1) showed different dynamics. Young (1.5–2 M) rats showed the highest FOSB/ΔFOSB immunoreactivity, which decreased in the elderly (18–24 M), without a peak in adolescence (1–3 M). These age-related changes in FOSB/ΔFOSB show high concordance with age-dependent dynamics of FOS (D’Costa et al., 1991; Kovács et al., 2018).

Upon ARS, PVN displays a constantly high FOSB/ΔFOSB signal thorough lifespan. This is in strong contrast with the dynamics of total PVN/FOS response that declined with the course of aging (Kovács et al., 2018). This age-dependent difference suggests that FOSB/ΔFOSB, and not the FOS response, may maintain the AP1-driven adaptive changes upon ARS in the PVN in old age. Because we recently saw that the FOSB/ΔFOSB response of PVN/CRH neurons decreases with age (Kovács et al., 2019), further studies must determine the identity of those PVN cells, which maintain and perhaps increase their reactivity in old age.

#### The Chronic Variable Mild Stress-Evoked FOSB/ΔFOSB Depends on Age in a Brain Area-Specific Manner

The CVMS increased the FOSB/ΔFOSB signal in three areas (BNSTfu, LHb, and PVN) in all examined age groups, which agrees with earlier studies using identical (Kovács et al., 2019) or very similar (Sterrenburg et al., 2011; Zhang et al., 2018) chronic stress models. We recently found that PVN/CRH neurons display a throughout-lifespan-constant FOSB/ΔFOSB reaction to CVMS (Kovács et al., 2019). In line with this, others (Herman et al., 2001), also, found that rats in an intermittent chronic stress model exhibit constant CORT response between 3 and 33 M. These data indicate that FOSB/ΔFOSB may be involved in the long-lasting regulation of the Crh gene (Kageyama et al., 2014), as the Crh gene promoter contains AP-1 sites (Malkoski and Dorin, 1999). Given the current results, the age-independent FOSB/ΔFOSB reaction might be not specific for CRH-cells of the PVN. Future studies must determine if other PVN cell populations (e.g., tyreotropin-releasing hormone-expressing cells) show a similarly constant response to CVMS.

Most of the examined nuclei (MeA, CeA, BNSTdm, BSNTv, BNSTfu, MHb, LHb, and DR) showed significant FOSB/ΔFOSB response to CVMS only in one or two of the examined age groups. This agrees with earlier reports regarding the BNSTov and CeA (Sterrenburg et al., 2011; Kovács et al., 2019). The distribution of these limited reactions shows two peaks: the first at the age of 6 months (CeA, MHb, LHb, DR) and the second at 18 months (BNSTdm, BNSTv, and BNSTfu). It seems that the CVMS-evoked FOSB/ΔFOSB is characteristic of adulthood and early senescence (6 and 18 M). This is in strong contrast with the age-related dynamics of ARS-induced FOSB/ΔFOSB response. Four regions (BLA, BNSTov, BNSTdl, and S1) did not respond to CVMS, which is in line with earlier studies (Sterrenburg et al., 2011; Kovács et al., 2019; Kang et al., 2021).

#### The Chronic Variable Mild Stress-Evoked FOSB/ΔFOSB Immunoreactivity Is Also a Function of Age

Ten nuclei (MeA, BLA, BNSTdl, BNSTdm, BNSTv, MHb, LHb, DR, cpEW, and S1) exhibited age-dependent dynamics in FOSB/ΔFOSB immunoreactivity. The highest FOSB/ΔFOSB signal was detected at 3 M in the BNSTdl, BNSTdm, BNSTv, EWcp, and DR. In the case of the MeA and S1, the maximum was detected at 2 M, while the habenulae (MHb, LHb) showed the peak in adulthood (6 M). All these nuclei displayed a decreasing activity beyond the peak with a minimum at 24 M. This dinamics is similar to that of FOS (Kovács et al., 2018) and FOSB/ΔFOSB (Kovács et al., 2019) observed in ARS and CVMS models, respectively.

In four nuclei (CeA, BNSTov, BNSTfu, and PVN), we did not detect any age-dependent change in the magnitude of the FOSB/ΔFOSB signal in CVMS animals. Interestingly, all these contain larger populations of CRH cells that also contribute to the control of stress responses (Choi et al., 2007; Herman et al., 2016). As CRH in cells displays decreasing FOSB/ΔFOSB signal during aging in the extended amygdala (Kovács et al., 2019), further studies are required to characterize the neurons that maintain the age-constant FOSB/ΔFOSB content and possibly contribute to the maintenance of stress response during aging.

### Age of Experimental Animals Does Matter

Age is a frequently overlooked variable in basic neuroscience. In most of the studies, young animals are used (2 months of age) and are erroneously referred to as “adults.” In contrast, this period represents late adolescence and early adulthood in the rat (McCutcheon and Marinelli, 2009; Sengupta, 2013). At this age, the maturation of the nervous system has not been completed yet. For instance, prefrontal parvalbumin, calretinin, and calbindin expression changes during the 3rd month of age, which corresponds to early adulthood (Caballero et al., 2014). A markedly accelerated axonal and synaptic development is characteristic of early puberty. Then, late adolescence and early adulthood are characterized by increased synaptic pruning (Crews et al., 2007), reorganization, and axon myelination (Brenhouse and Andersen, 2011; Green and McCormick, 2013). Our present results and previous observations (Kovács et al., 2018, 2019) also support that the maximum FOS and FOSB/ΔFOSB response is characteristic of these age periods. Nevertheless, PVN/CRH cells showed the highest FOS or FOSB/ΔFOSB response in the pre-pubertal period (1 M) characterized by prolonged ACTH and CORT response compared to late adolescence (Eiland and Romeo, 2013).

The ARS-induced PVN FOS reactivity peaks at 2 M (Kovács et al., 2018), which later declines and mirrors age-dependent reactivity in the stress response. In contrast, in this study using a different activation marker FOSB/ΔFOSB, we saw a stable and age-independent response to ARS. This suggests that the use of an age-independent activation marker might be beneficial if relatively young animals are used in an experiment.

Because semi-quantitation of IEG expression is also used beyond the field of stress research (Eagle et al., 2018), considering the fast-changing plasticity of the central nervous system in adolescence and young adulthood (Crews et al., 2007; McCutcheon and Marinelli, 2009; Eiland and Romeo, 2013), precise planning of experiment concerning the age of animals, is also essential.

### Limitations

The antibody used here does not differentiate the N-terminal epitope of full-length FOSB from the ΔFOSB. In areas characterized by strong basal FOSB/ΔFOSB (e.g., nuclei of the extended amygdala and habenula), we presumably detect both proteins. In the ARS model, we sacrificed the animals 2 h after the onset of the stimulus. Therefore, here, we detected mainly the *de novo*-produced full-length FOSB, since it appears earlier and in a larger amount than the ΔFOSB does (Nestler, 2015). In contrast, CVMS animals were terminated 24 after the last stress exposure. Therefore, the FOSB/ΔFOSB signal should correspond to ΔFOSB because the full-length FOSB has already disappeared at this time (Nestler, 2015).

Areas involved in the processing of sensory information and memory consolidation (e.g., amygdala nuclei) exhibited high basal FOSB/ΔFOSB immunoreactivity that did not change upon stress. In these regions, it is plausible that the ratio of full-length FOSB and ΔFOSB has altered, but our antibody cannot help to prove this.

Another important limitation is that we do not know the functional identity of FOSB/ΔFOSB-containing neurons, therefore, it is hard to predict their inhibitory or excitatory (Bowers et al., 1998; Choi et al., 2007) role in stress adaptation; moreover, some inhibitory types of neurons (Reisch et al., 2007) do not show remarkable IEG expression.

In the CVMS model, fewer nuclei showed remarkable activity change than in the ARS model. CVMS is a widely used model for mood disorders, but it is also not without limitations as the sensitivity to CVMS might be brain area-specific, and we also cannot exclude that some brain regions (e.g., divisions of the extended amygdala) have already adapted to some extent to the stressors that limited their FOSB/ΔFOSB activation. Nevertheless, we preferred the CVMS model because, due to the unpredictability of stressors, animals adapt to a lesser extent, in contrast to homotypic chronic stress models (Bali and Jaggi, 2015; Czéh et al., 2016). This is well-demonstrated by physiological parameters of our rats [i.e., adrenal- thymus- and bodyweight, CORT levels (see in ref. Kovács et al., 2019)], indicating higher HPA-axis activity in CVMS.

The age-dependent decline of FOS and FOSB/ΔFOSB in most of the examined nuclei may refer to the reduced amount of AP-1 (Chen et al., 1997; Kovács, 1998), thus, suggesting that functions controlled by AP-1 might also be age-dependent. Because the abundance of JUN proteins does not decrease in senescence (Smith et al., 2001), it is highly plausible that the AP-1 activity decreases with age (Asanuma et al., 1995; Smith et al., 2001; Ryan et al., 2015). This assumption, however, requires further testing because of the following: (a) the AP-1 may be formed by various members of FOS and JUN families (Hope et al., 1992; Okuno, 2011); (b) JUND and ΔFOSB proteins may form homodimers with transcriptional activity (Ryseck and Bravo, 1991); (c) the composition of AP-1 is subject to change with time after stress (Sonnenberg et al., 1989; Hope et al., 1992); and (d) the composition of AP-1 is also age-dependent (Asanuma et al., 1995).

The primary somatosensory (S1) cortex was included in this study to test whether the aging-related decline in the sensitivity of the sensory systems might have contributed to the reduction of FOSB/ΔFOSB response. In our earlier work we saw that the FOS response to ARS shows U-shaped dynamics, suggesting that the aging-related deterioration does not explain the reduction of FOS response (Kovács et al., 2018). Although in this study we saw some limited decline of the FOSB/ΔFOSB signal’s CVMS response with age, the magnitude of the decline and the high basal expression suggests that the sensory systems were not severely compromised in our rats. The decline might be attributable in part to the aging-related cortical atrophy (Yang et al., 2021) also.

A deeper understanding of sex differences in the brain’s stress sensitivity at the IEG expression level (Sterrenburg et al., 2011, 2012; Girard-Joyal et al., 2015; Baum et al., 2018; Worley et al., 2020) and coping strategies might help to understand the neurobiological background of well-known gender differences in the epidemiology of mood disorders. Therefore, it would have been interesting to expand this study to a cohort of female rats. Nevertheless, considering the capacity limitations and the estrus cycle phase as an additional factor in fertile female animals in IEG response (Egan et al., 2018), this comparison was not realistic. Therefore, it is a true limitation of this study that our findings may not apply to the female brain.

## Conclusion

Concerning the limitations, to the best of our knowledge, this is the only systemic description of FOSB/ΔFOSB immunoreactivity in control, ARS, and CVMS-exposed male rats throughout the lifespan. The highest ARS-evoked FOSB/ΔFOSB content in the examined nuclei was found in late adolescence-early adulthood (1.5–3 M). The maximum of FOSB/ΔFOSB response to CVMS was less characteristic for a life period and varied between 2 and 12 M in the examined brain areas.

The PVN and BNSTfu did not exhibit an age-dependent FOSB/ΔFOSB decline upon ARS and CVMS from early adulthood (2–3 M) to the senescence (18–24 M). The magnitude of FOSB/ΔFOSB’s rise is age- and stressor-related. The age-associated dynamics of FOSB/ΔFOSB increase are different from the FOS, which indicates the possible difference in their function. Our findings further support that the magnitude of the neuronal stress response is age and brain-area-dependent also at the level of IEGs.

## Data Availability Statement

The raw data supporting the conclusions of this article will be made available by the authors, without undue reservation.

## Ethics Statement

The animal study was reviewed and approved by the Animal Welfare Committee of Pécs University, the National Scientific Ethical Committee on Animal Experimentation in Hungary, and the National Food Chain Safety Office in Hungary.

## Author Contributions

LK evaluated the results, performed statistics, prepared the figures, and wrote the draft manuscript. BU, NF, and AG performed animal experiments, immunolabeling, digital imaging, and cell counting. BG designed the experiments, collected the blood samples, performed perfusion, supervised the tissue preparation, imaging, selected the images containing the areas of interest, helped with figure preparation, and supervised the manuscript. All authors contributed to the article and approved the submitted version.

## Conflict of Interest

The authors declare that the research was conducted in the absence of any commercial or financial relationships that could be construed as a potential conflict of interest.

## Publisher’s Note

All claims expressed in this article are solely those of the authors and do not necessarily represent those of their affiliated organizations, or those of the publisher, the editors and the reviewers. Any product that may be evaluated in this article, or claim that may be made by its manufacturer, is not guaranteed or endorsed by the publisher.

## Footnotes

1
https://www.abcam.com/fos-b-antibody-epr15905-ab184938.html#lb
